# Tip-enhanced nanocavities amplify the sum frequency generation

**DOI:** 10.1038/s41377-025-01946-3

**Published:** 2025-08-22

**Authors:** Chun-Chieh Yu, Yuancheng Jing, Wei Xiong

**Affiliations:** 1 Department of Chemistry, University of California, San Diego, La Jolla, CA 92093 USA; 2 Materials Science and Engineering Program, University of California, San Diego, La Jolla, CA 92093 USA; 3 Department of Electrical and Computer Science Engineering, University of California, San Diego, La Jolla, CA 92093 USA

**Keywords:** Nanophotonics and plasmonics, Infrared spectroscopy

## Abstract

Tip-enhanced vibrational sum frequency generation (VSFG) spectroscopy is proposed and demonstrated. Incorporation with the plasmon cavities leads to significant signal amplification—up to 14 orders of magnitude.

Vibrational sum frequency generation (VSFG), a second-order nonlinear process, is a powerful tool to study surface chemistry^1,2^. VSFG occurs when visible and infrared (IR) light beams spatially and temporally overlap at a centrosymmetry-broken system, including a surface or an interface (see Fig. 1a). When the IR frequency is resonant to the molecular vibrational mode, the VSFG signal is enhanced. Thus, VSFG signal provides the surface- and molecular-specific information and has offered molecular insights into various interfacial systems, such as air/water^1,3,4^, semiconductor^5–8^, and biological interfaces^9,10^. Yet, most of molecular samples have weak second-order susceptibilities, making the VSFG process difficult. As such, a laser source with high peak power, usually a high-power picosecond or femtosecond pulsed laser, along with optical parametric amplification to generate pulsed IR beams, is required for the conventional VSFG experiment^11^.

**Fig. 1:**
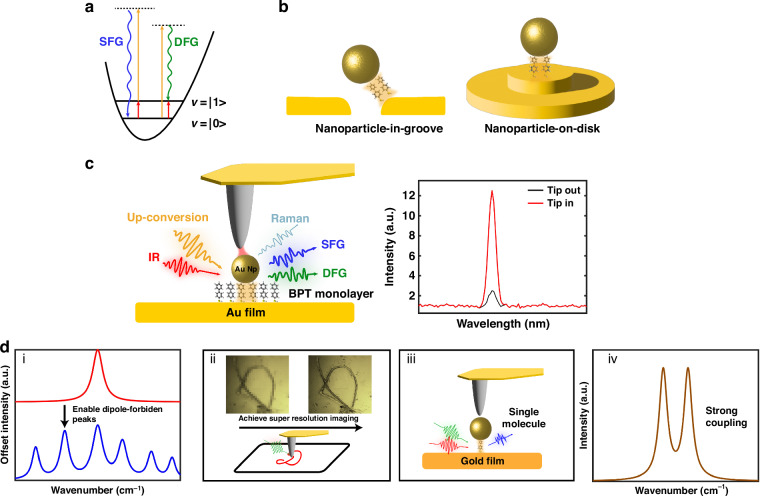
Nanocavities enhance sum frequency generation. **a** Jablonski energy diagram of sum frequency generation (SFG) and difference frequency generation (DFG). The blue arrow represents the SFG. The green arrow represents the DFG. The red and orange arrows represent the IR and visible lights, respectively. **b** Two kinds of modified nanoparticle-on-mirror (NPoM) cavity structures^12,13^. **c** Tip-enhanced NPoM cavity. When the metal tip is close to the gold nanoparticle, the SFG signal will be enhanced, as illustrated in the right panel. **d** Schematic of the prospect of Tip-enhanced VSFG. (i) Tip modification effect on the dipole-forbidden vibrational modes. (ii) Super-resolution VSFG image. (iii) Single-molecular VSFG response (iv) Controlling the Strong light-matter coupling

In 2021, significant advancements were reported by two independent groups^12,13^. Both groups successfully demonstrated highly efficient VSFG upconversion processes using continuous-wave (CW) lasers. The key is incorporating molecules inside the modified nanoparticle-on-mirror (NPoM) cavity structures (see Fig. 1b)^14^. Specifically, the Galland group utilized a nanoparticle-in-groove cavity configuration^12^, whereas the Baumberg group used a nanoparticle-on-disk structure^13^. Despite the difference in cavity structure, both molecular nanocavities are dedicatedly designed to resonant at both the IR and visible frequencies. This double-resonant mechanism enhances the upconversion efficiency by exceeding 10 orders of magnitude, and allows the VSFG upconversion to be performed using microwatt-level CW lasers.

Recently, in *Light: Science & Applications*, Philippe Roelli, Rainer Hillenbrand, and coworkers reported an innovation of this approach^15^. Inspired by the scattering-type scanning near-field optical microscope (s-SNOM) that the tip field modulates the local field environment^16^, the authors found that placing a metallic tip above the NPoM cavity could similarly enhance the SFG upconversion, without the need for the IR-resonant cavity structure (see Fig. 1b, c). While the NPoM cavity enhances the electromagnetic fields in the visible regime, the tip further boosts the IR local fields—leading to a significant signal amplification—up to 14 orders of magnitude. The authors systematically examined the tip-position dependence of the VSFG enhancement, finding that significant enhancement occurred only when the tip was positioned on the cavity side illuminated by both visible and IR beams. Conversely, no enhancement occurred when the tip was placed on the opposite side. The asymmetric response is attributed to the tip response to the longitudinal and transversal directions under the visible light illumination. Additionally, the authors showed that tuning the tip height can easily control the VSFG enhancement, which implies the ability to adjust the coupling between the cavity and molecules precisely.

This work is unique in that it is the first time combining the molecule-filled NPoM cavities with the near-field microscope, enabling on-demand control of the IR enhancement and simultaneous acquisition of spectral and spatial information. This capability is essential to explore the molecular adsorption and molecular self-assembly process on the surface^17,18^. Moreover, this approach offers a more versatile platform, requiring no modification of the NPoM cavities, and thus circumventing the previously necessary complex design and fabrication processes^12,13^. Furthermore, by nanopositioning the metal tip, the significant VSFG signal enhancement, even under low-power CW laser radiations, opens possibilities for exploring the VSFG response to a single-molecular level.

This work reduces the constraints typically imposed on VSFG samples and extends the application of VSFG to a broader scientific community. The tip-enhanced SFG enables the investigation of samples with weak VSFG signals and the unnecessary need for large sample sizes. Moreover, this approach allows for probing the VSFG signal below 1000 cm⁻^1^, thereby paving the way to explore low-frequency vibrational modes. Nevertheless, this technique still has considerable potential for further investigation (see Fig. 1d). For example, how does the tip modification affect the VSFG signal? How does the polarization of incident light impact the VSFG enhancement? How much spatial resolution can it achieve? It may be one way to accomplish super-resolution VSFG imaging. Is it possible to extend this method to the electronic SFG? Additionally, it would be of great interest to combine this method with other techniques like heterodyne detection for the phase information or time-resolved SFG technique for probing energy transfer dynamics^19–23^. Finally, its potential to reach strong light-matter coupling and single-molecule nonlinear optics opens new routes for quantum optics of molecular sciences.
